# Biosignature for airway inflammation in a house dust mite-challenged murine model of allergic asthma

**DOI:** 10.1242/bio.014464

**Published:** 2016-01-06

**Authors:** Hadeesha Piyadasa, Anthony Altieri, Sujata Basu, Jacquie Schwartz, Andrew J. Halayko, Neeloffer Mookherjee

**Affiliations:** 1 Manitoba Centre for Proteomics and Systems Biology, University of Manitoba, Winnipeg, Manitoba, R3E 3P4, Canada; 2 Department of Immunology, University of Manitoba, Winnipeg, Manitoba, R3E 0T5, Canada; 3 Department of Physiology and Pathophysiology, University of Manitoba, Winnipeg, Manitoba, R3E 0J9, Canada; 4 Biology of Breathing Group, Children's Hospital Research Institute of Manitoba, Winnipeg, Manitoba, R3E 3P4, Canada; 5 Department of Internal Medicine, University of Manitoba, Winnipeg, Manitoba, R3T 2N2, Canada; 6 Canadian Respiratory Research Network

**Keywords:** Allergy, Asthma, Biosignature, Biomarkers, House dust mite, Inflammation, Airway hyper-responsiveness

## Abstract

House dust mite (HDM) challenge is commonly used in murine models of allergic asthma for preclinical pathophysiological studies. However, few studies define objective readouts or biomarkers in this model. In this study we characterized immune responses and defined molecular markers that are specifically altered after HDM challenge. In this murine model, we used repeated HDM challenge for two weeks which induced hallmarks of allergic asthma seen in humans, including airway hyper-responsiveness (AHR) and elevated levels of circulating total and HDM-specific IgE and IgG1. Kinetic studies showed that at least 24 h after last HDM challenge results in significant AHR along with eosinophil infiltration in the lungs. Histologic assessment of lung revealed increased epithelial thickness and goblet cell hyperplasia, in the absence of airway wall collagen deposition, suggesting ongoing tissue repair concomitant with acute allergic lung inflammation. Thus, this model may be suitable to delineate airway inflammation processes that precede airway remodeling and development of fixed airway obstruction. We observed that a panel of cytokines e.g. IFN-γ, IL-1β, IL-4, IL-5, IL-6, KC, TNF-α, IL-13, IL-33, MDC and TARC were elevated in lung tissue and bronchoalveolar fluid, indicating local lung inflammation. However, levels of these cytokines remained unchanged in serum, reflecting lack of systemic inflammation in this model. Based on these findings, we further monitored the expression of 84 selected genes in lung tissues by quantitative real-time PCR array, and identified 31 mRNAs that were significantly up-regulated in lung tissue from HDM-challenged mice. These included genes associated with human asthma (e.g. *clca3*, *ear11*, *il-13*, *il-13ra2*, *il-10*, *il-21*, *arg1* and *chia1*) and leukocyte recruitment in the lungs (e.g. *ccl11*, *ccl12* and *ccl24*). This study describes a biosignature to enable broad and systematic interrogation of molecular mechanisms and intervention strategies for airway inflammation pertinent to allergic asthma that precedes and possibly potentiates airway remodeling and fibrosis.

## Abstract (precis)

**Summary:** This study describes a systematic analysis of molecular end points in an murine model of allergic asthma. The biosignature described can be used to interrogate molecular mechanisms and intervention strategies for airway inflammation pertinent to allergic asthma that precedes and possibly potentiates airway remodeling and fibrosis.

## INTRODUCTION

Allergic asthma is a common chronic inflammatory lung disease, affecting nearly 300 million people worldwide, with significant health, health service and economic burden (www.publichealth.gc.ca). The disease is primarily driven by repeated exposure to allergens and characterized by airflow obstruction, airway hyper-responsiveness (AHR) and airway inflammation. Lung inflammation in asthma is typically orchestrated by allergen-driven activation of innate immune cells followed by an exacerbated Th2-biased inflammation and synthesis of allergen-specific IgE antibody, which initiates the release of inflammatory mediators from immune cells (Busse and Lemanske, 2001; Jacquet, 2011). As asthma is a heterogenous disorder with different sub-phenotypes (e.g. Th2-High vs Th2-Low; Woodruff et al., 2009), there are no therapies that are effective in all asthma patients. This has contributed to there being rather modest progress in developing new therapies that have a broad impact. Animal models of human asthma have not been extensively characterized using a Systems Biology approach, which has created a knowledge gap that has greatly limited success in promoting development of new therapies.

House dust mite (HDM), *Dermatophagoides* spp., is associated with allergic response in up to 85% of asthma patients worldwide (Gregory and Lloyd, 2011; Gandhi et al., 2013). Thus, in the last decade, HDM-challenged murine models have been used to dissect different aspects of the pathogenesis and to begin to define some of the molecular mechanisms that may be important in the disease process of allergic asthma (Stevenson and Birrell, 2011). These models involves the sensitization of the animal to HDM by repeated intranasal challenge which results in a Th2-polarized bronchial inflammation, airway remodeling and epithelial damage similar to that seen in human asthma (Cates et al., 2004, 2007; Johnson et al., 2004). The advantage of this model, in contrast to the commonly used ovalbumin-exposure murine models, is that HDM is a natural inhaled antigen and repeated exposure to HDM is not associated with the development of tolerance (Cates et al., 2004). Previous studies have shown that repeated HDM exposure of two to three weeks, considered to be acute exposure, induces markedly mixed (eosinophilic and neutrophilic) airway inflammation and AHR to methacholine challenge (Cates et al., 2004). Whereas, mice subjected to repeated HDM exposure for five to eight weeks (the chronic HDM challenge model) results in airway inflammation along with significant airway wall remodeling, including airway smooth muscle, epithelial and goblet cell hyperplasia, accumulation of collagen, fibronectin and other extracellular matrix proteins that manifest as airway wall fibrosis and thickening (Locke et al., 2007).

A major challenge in using the HDM-challenged murine model is that the immune responses and physiological outcomes vary depending on the sensitization protocol and the time point at which the animals are sacrificed after the last HDM challenge. Moreover, studies that use systematic appraisal of how individual pathways, biological mediators and cells contribute in an integrated manner to specific aspects of the disease phenotype are lacking. For example, neutrophils are detected relatively early (Monteseirin, 2009; Al Heialy et al., 2011) after HDM exposure with peak numbers evident in the bronchoalveolar lavage fluid (BALF) 6-12 h (De Alba et al., 2010). In contrast, peak numbers of lung eosinophils occurs beyond 24 h and observed at 48 h after last HDM challenge (De Alba et al., 2010). Despite the use of the HDM-challenge in mice as a preclinical model for asthma, very few studies have comprehensively characterized the immune responses and identified specific biomarkers that can be objectively used to monitor disease progression or predict responses to candidate therapeutics (Ho et al., 2014; Koyama et al., 2015).

In this study we used the acute (2-week) HDM-challenge model murine model to characterize changes in the expression of 84 genes associated with allergy and asthma, using a quantitative real-time PCR (qPCR) array. We also employed a multiplex cytokine profiling platform to define specific cytokine responses in the lung tissues, BALF and serum, in the HDM-challenged mice. We analyzed the data in the context of our observations that AHR develops only after an initial burst of inflammation (up to 8 h). Thus, we focused on examining the physiological outcomes and defining a biosignature of transcripts 24 h after the last HDM challenge, a time point between peak neutrophilic and eosinophilic inflammation. The acute model of HDM-challenge described in this study generated airway inflammation and AHR, preceding airway remodeling and fibrosis. Therefore, we speculate that the panel of quantitated protein and mRNA endpoints described in this study will be surrogates for the human disease, and can be used to interrogate molecular mechanisms and intervention strategies in airway inflammation that precedes and possibly potentiates airway remodeling and fibrosis.

## RESULTS

### Kinetics of cellular infiltration in the BALF following HDM challenge

**Fig. 1.**
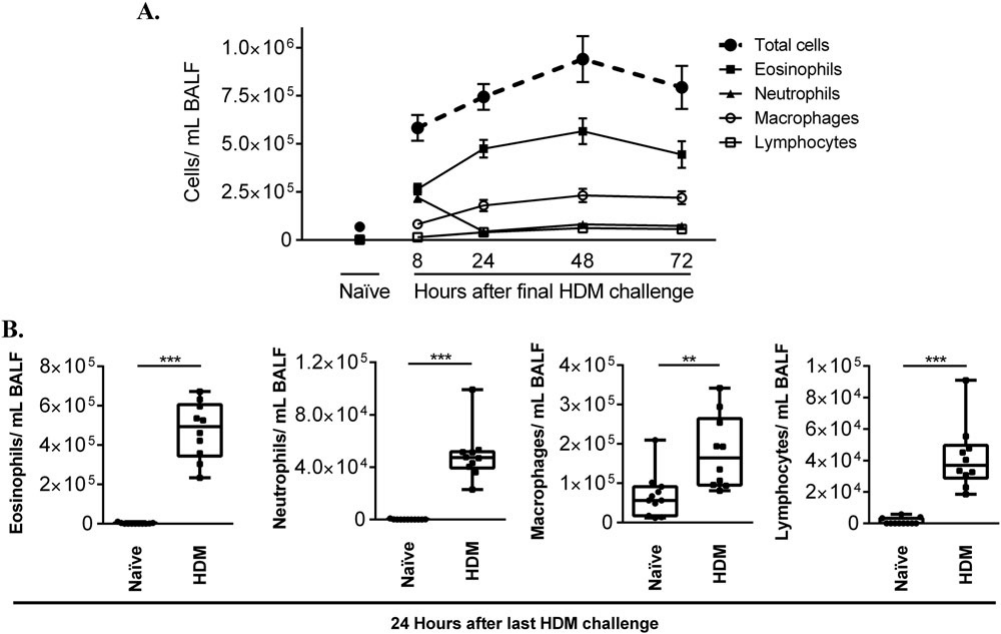
**HDM challenge increases immune cell infiltration to lungs.** 8-10-week-old female Balb/c mice were challenged by intranasal administration of 35 µl of whole HDM extract (0.7 mg/ml) in saline, for 2 weeks. Broncheoalveolar lavage fluid (BALF) was collected 8, 24, 48 and 72 h after last HDM challenge, and the supernatant and cell fractions were separated by cytospin. Inflammatory cells were stained using a modified Wright-Giemsa staining (Hema 3^®^ Stat Pack). Individual cell populations were measured in 8 image frames and counted by 2 different blinded personnel. (A) Kinetics of infiltration of eosinophil, neutrophil, macrophage and lymphocyte population, and (B) cell infiltration assessed 24 h after last HDM challenge, in HDM-challenged (*n*=10) compared to allergen-naïve mice (*n*=10). Mann–Whitney *U*-test was used to assess statistical significance (**P≤0.005, ***P≤0.0005). Error bars shown represent s.e.m.

To assess the kinetics of lung inflammation, BALF samples collected 8, 24, 48 and 72 h after the last HDM challenge were used for cell differential analyses. Peak neutrophil infiltration was observed 8 h after the last HDM challenge, which subsequently rapidly declined (Fig. 1A). However, eosinophil and macrophage infiltration was observed beyond 8 h, and steadily increased peaking at 48 h after the last HDM challenge (Fig. 1A). There was a steady increase of total lymphocyte population beyond 8 h to 72 h after last HDM challenge (Fig. 1A). All the cell types significantly increased in the BALF 24 h after the last HDM challenge, in the HDM-challenged mice compared to allergen-naïve mice (Fig. 1B).

### HDM-challenged mice exhibit AHR

**Fig. 2.**
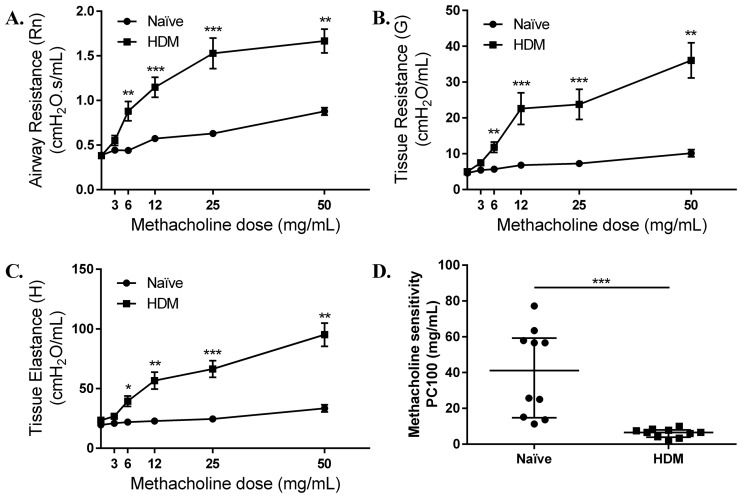
**HDM challenge increases airway hyper-responsiveness.** 8-10-week-old female Balb/c mice were challenged by intranasal administration of 35 µl of whole HDM extract (0.7 mg/ml) in saline, for 2 weeks. Lung mechanics of naïve (*n*=10) and HDM-challenged (*n*=10) were monitored using a *flexi*Vent™ small animal ventilator, 24 h after the last HDM challenge. Baseline airway and tissue resistance and tissue elastance was calculated using saline. Mice were exposed to increasing dose of methacholine (3-50 mg/ml) and the change in (A) airway resistance, (B) tissue resistance and (C) tissue elastance was monitored. (D) Central airway sensitivity to methacholine was measured by calculating concentration of methacholine required to double baseline resistance (PC100). Mann–Whitney *U*-test was used for statistical analyses (*P≤0.05, **P≤0.005, ***P≤0.0005). Error bars shown represent s.e.m.

Previous studies using different HDM-sensitization protocols and monitored at different time points after the last HDM-challenge, have all demonstrated that HDM-challenge results in significant AHR (Lewkowich et al., 2008; Saglani et al., 2009; van der Velden et al., 2014). We monitored lung function in our specified acute model at 8 and 24 h after last HDM challenge to confirm that the sensitization protocol induced AHR after two weeks of HDM-challenge. To that end, lung function was measured using the *flexi*Vent™ small animal ventilator in response to inhaled methacholine (0 to 50 mg/ml). HDM-challenged mice exhibited a significant increase in maximum airway resistance (Fig. 2A), tissue resistance (Fig. 2B) and tissue elastance (Fig. 2C) when measured 24 h after the last HDM-challenge compared to allergen-naïve mice. These responses though significant were not robust when measured 8 h after last HDM challenge (Fig. S1). Furthermore, hypersensitivity to inhaled methacholine was elevated for airway resistance after HDM-challenge, as indicated by a significant reduced methacholine PC100 (amount required to double the baseline resistance), 24 h after the last HDM challenge (Fig. 2D).

### Two weeks of HDM-challenge results in early signs of airway tissue remodeling, without collagen deposition

**Fig. 3.**
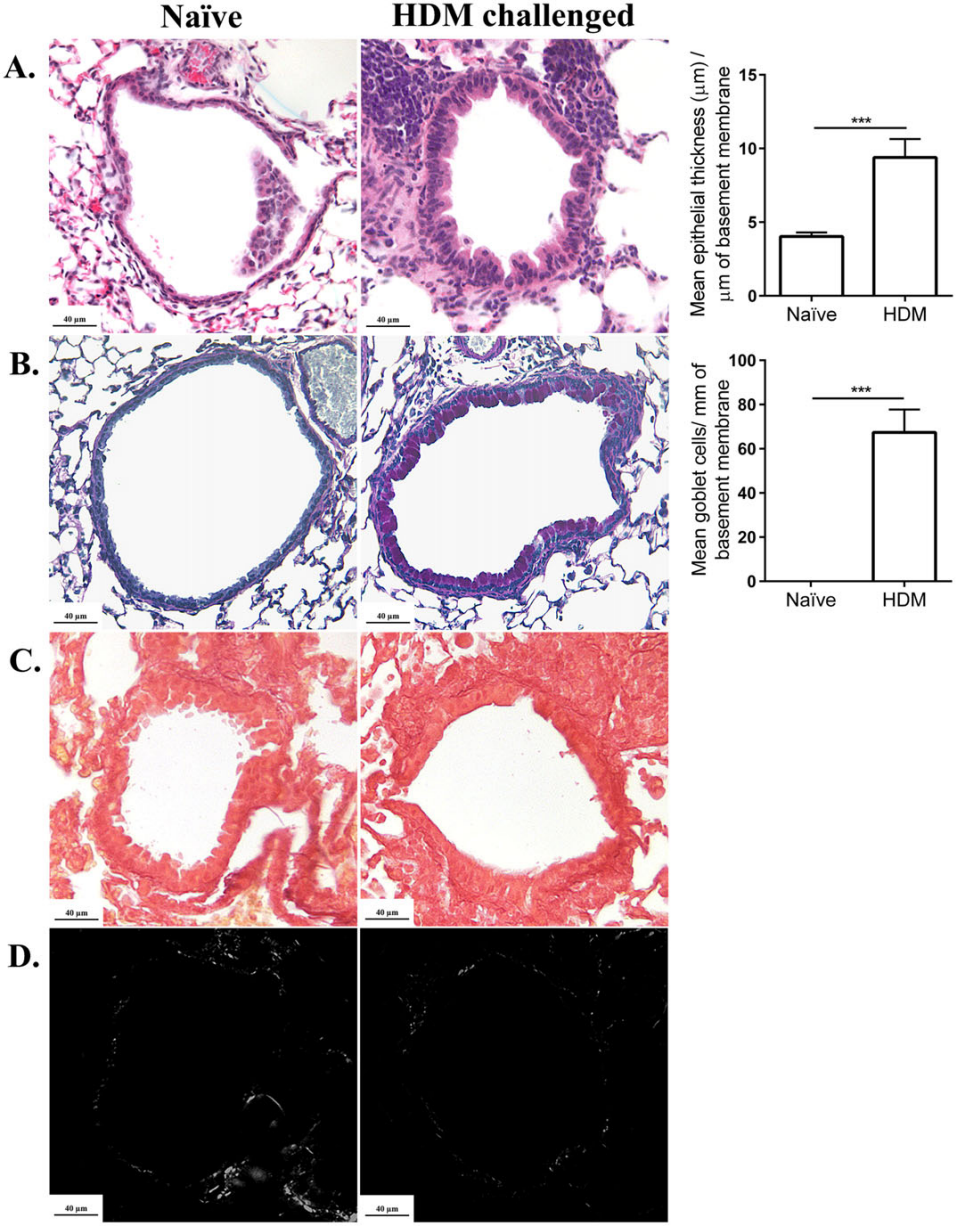
**Histological assessment of lung sections.** 8-10-week-old female Balb/c mice were challenged by intranasal administration of 35 µl of whole HDM extract (0.7 mg/ml) in saline, for 2 weeks. (A,B) Paraffin embedded lung sections (6 µm) were stained with (A) hematoxylin and eosin (H&E) to enumerate cell infiltration and epithelial thickening, (B) periodic acid Schiff (PAS) to assess goblet cells, in HDM-challenged (*n*=10) compared to allergen-naïve (*n*=10) mice. Right-hand panels: quantification of mean epithelial thickness and goblet cells/mm of the basement membrane. Mann–Whitney *U*-test was used for statistical analyses (***P≤0.0005). Error bars shown represent s.e.m. (C,D) Picrosirius Red stain was used to enumerate collagen deposition viewed using (C) bright or (D) polarized contrast, in light microscopy.

To correlate changes in lung inflammation (Fig. 1) and lung function (Fig. 2) with the status of tissue repair, we performed complementary assessment of cell infiltration into tissues surrounding the airways using histology. Consistent with the cell differential (Fig. 1B), H&E staining of the lung sections of HDM-challenged mice showed significant cellular influx to the peribronchial and perivascular area, and semi-quantitative analysis showed that there was significant increase in epithelial thickness in the HDM-challenge compared to allergen-naïve mice (Fig. 3A). PAS stain showed marked accumulation of mucous secreting goblet cells in the epithelium of HDM-challenged mice and semi-quantitative showed that there was significant increase in the number of goblet cells in HDM-challenged mice compared to the allergen-naïve mice (Fig. 3B). Finally, to determine whether our HDM-challenge protocol induced structural airway remodeling, we performed Picrosirius staining to assess collagen deposition, which is a hallmark of airway remodeling in asthma (Vignola et al., 2001). Picrosirius staining revealed that there was no marked change in collagen deposition and evidence of subepithelial fibrosis after two week of HDM-challenge (Fig. 3C and D).

### Serum levels of total and HDM-specific IgE and IgG are significantly elevated in the HDM-challenged mice

**Fig. 4.**
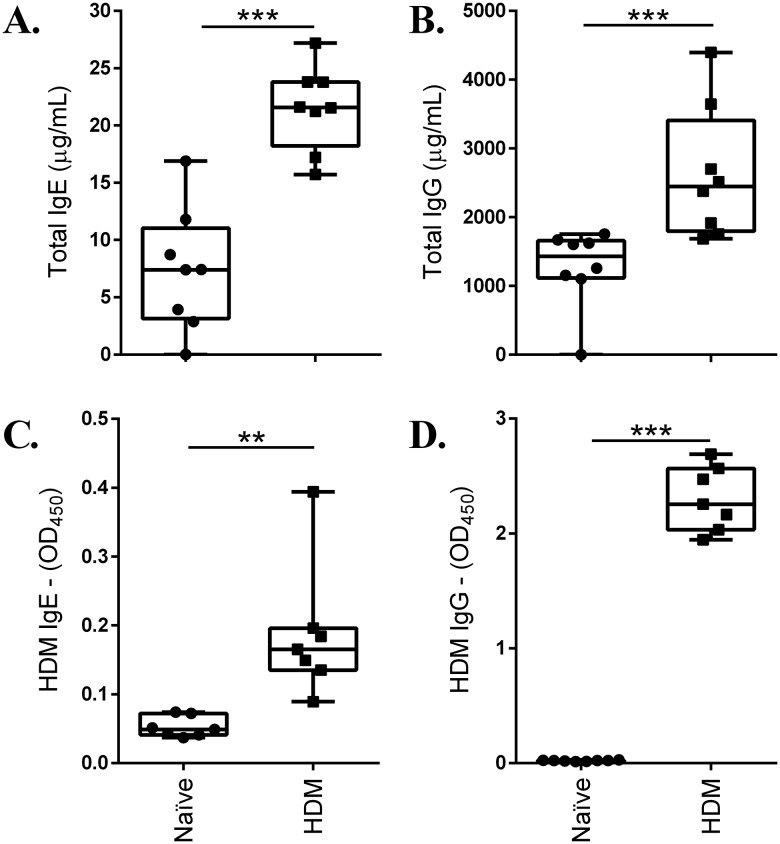
**HDM challenge increases immunoglobulin levels.** 8-10-week-old female Balb/c mice were challenged by intranasal administration of 35 μl of whole HDM extract (0.7 mg/ml) in saline, for 2 weeks. Immunoglobulin levels, (A) total IgE, (B) total IgG, (C) HDM-specific IgE and (D) HDM-specific IgG1, were monitored in serum from naïve (*n*=8) and HDM-challenged (*n*=8) mice, 24 h after the last HDM challenge, by ELISA. Results are shown as box plots with median line, and statistical analyses was performed using the Mann-Whitney U-test (**P≤0.005, ***P≤0.0005).

HDM-challenged mice showed significantly higher serum levels of total IgE (Fig. 4A) and IgG (Fig. 4B), as well as HDM-specific IgE (Fig. 4C) and IgG1 (Fig. 4D), compared to allergen-naïve mice.

### HDM challenge significantly alters gene expression in the lungs

There are limited studies that include comprehensive analyses of transcriptional responses from murine lung following HDM-challenge. In order to provide objective readouts in this model, we used a disease-focused PCR array to monitor the expression of selected 84 genes related to allergy and asthma in HDM-challenged (*n*=3) compared to allergen naïve (*n*=3) mice (the entire data set is provided as Table S1). Differentially expressed (DE) genes were defined as those that were either up- or downregulated by ≥2-fold with associated *P*≤0.05, in HDM-challenged compared to naïve mice. Our analyses revealed marked changes in the gene expression profile of lungs in the HDM-challenged compared to allergen-naïve mice (Fig. S2). The abundance of transcripts for 39% of genes (34 out of 84 genes) monitored was differentially expressed after HDM-challenge (Table 1). Of the DE genes, 31 were increased by >2-fold, whereas only three transcripts were downregulated after HDM-challenge (Table 1). Among the transcripts that were increased after HDM-challenge, most markedly induced were those for the chloride channel calcium activated member 3 (*clca3*), Eosinophil associated ribonuclease A family 11 (*ear11*) and Mucin-5AC (*Muc5ac*), each of these has been previously identified as potential biomarkers in asthma (Laprise et al., 2004; Woodruff et al., 2007; Park et al., 2008; Louten et al., 2012). As expected, Th2-driven cytokines and related receptors (*il-13*, *il-13ra2*, *il-4*), chemokines (*ccl11*, *ccl12 and ccl24*), and genes associated with allergic diseases arginase 1 (*arg1*) and chitinase (*chia1*) (Woodruff et al., 2007; Lloyd and Hessel, 2010; Zhu, 2010) were also significantly upregulated in the HDM-challenged compared to allergen-naïve lungs (Table 1).

**Table 1.**
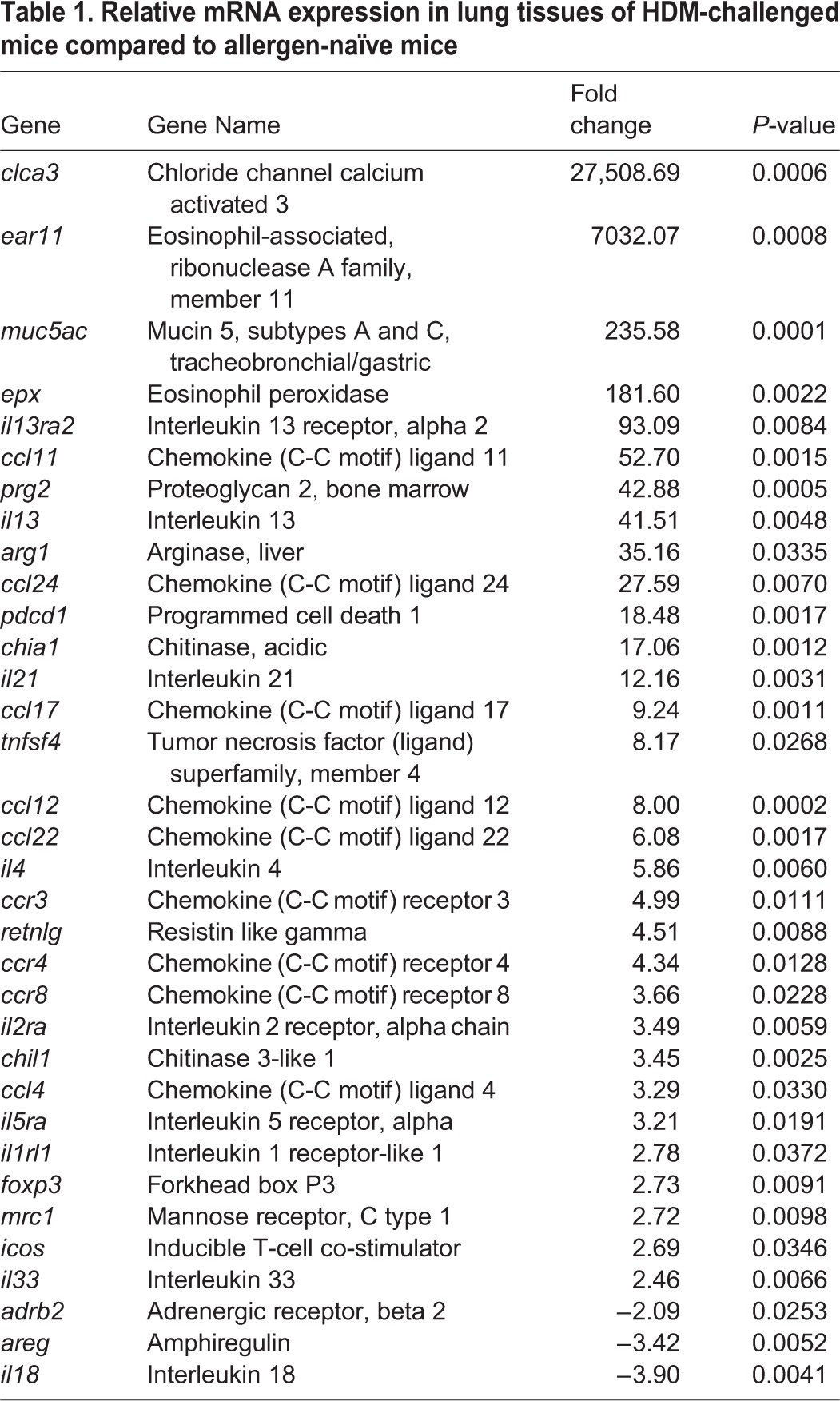
**Relative mRNA expression in lung tissues of HDM-challenged mice compared to allergen-naïve mice**

**Fig. 5.**
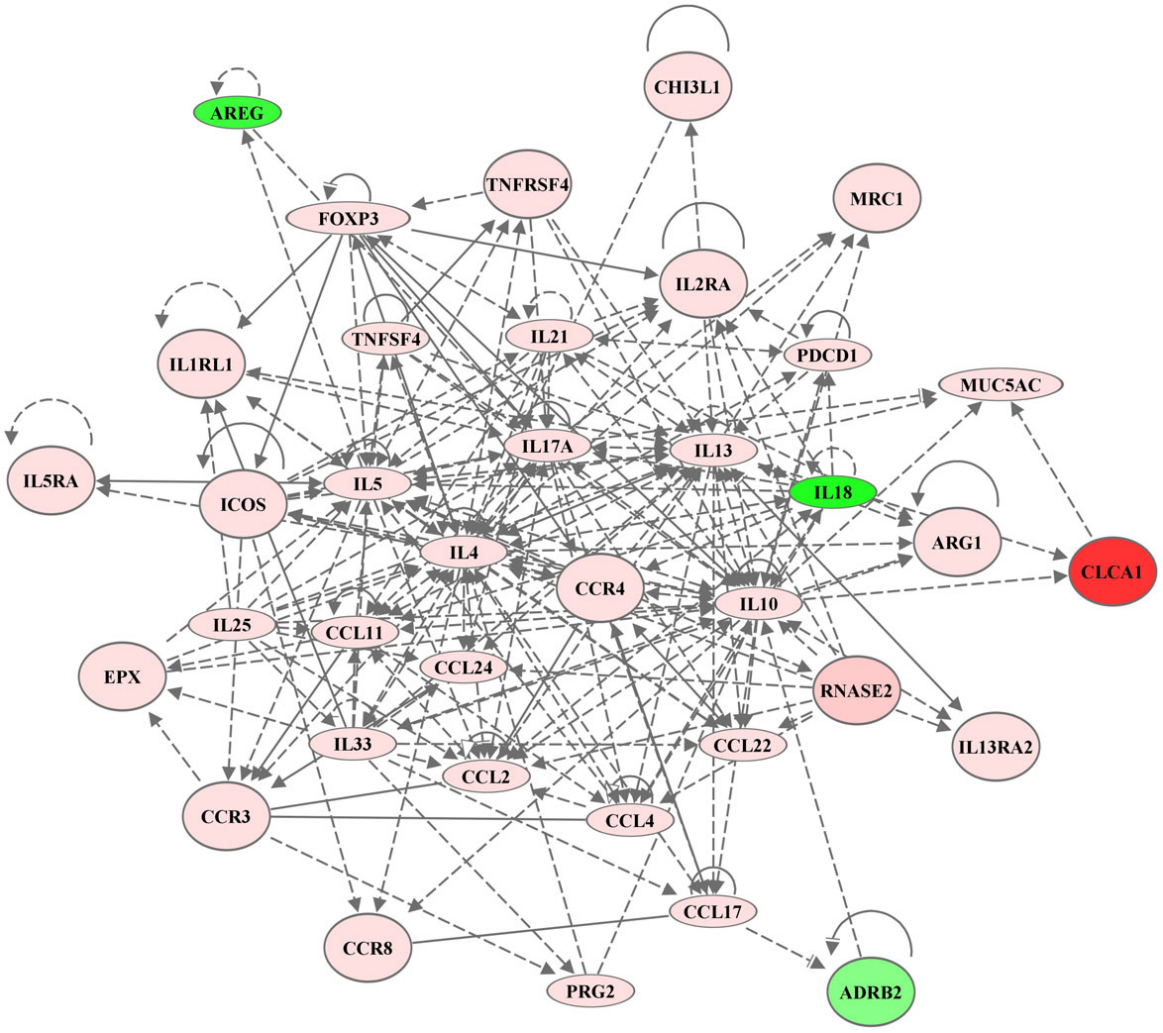
**Interaction between the genes differentially expressed in response to HDM.** Of the 34 DE genes in HDM-challenged lung tissues (Table 1), 33 connected in an interaction network using the Ingenuity^®^ Pathway Analysis tool. Red nodes represent upregulated and green nodes represent downregulated genes. The intensity of color corresponds to the magnitude of fold change in HDM-challenged lungs relative to that in allergen-naïve mice. Solid lines correspond to direct interactions and dotted lines correspond to indirect interactions.

We further interrogated the DE genes using Ingenuity^®^ Pathway Analysis (IPA) tool to assess biological processes, interactions and upstream regulators in the data set. Of the 34 DE genes, 33 genes were connected by network analyses employing both direct and indirect relationship (Fig. 5). The IPA upstream regulator analytic identified 12 upstream transcriptional regulators known to activate the DE genes, and nine regulators known to inhibit the DE genes (Table S2; Fig. S3A). Furthermore, the mRNA expressions of some of the predicted upstream regulators notably *il-33*, *il-13* and *il-4* were also upregulated in the lungs of HDM-challenged compared to allergen-naïve mice (Table 1; Fig. S3B). Predominant biological processes predicted to be activated with high confidence as a consequence of the DE genes were AHR, leukocyte migration, granulocyte adhesion and diapedesis, differentiation of leukocytes, T-cell helper differentiation, cytokines and chemokines signaling, flux of calcium and secretion of mucus, all known to be involved in inflammation and hypersensitivity.

### HDM challenge selectively alters cytokine and chemokine profile in the lungs

**Fig. 6.**
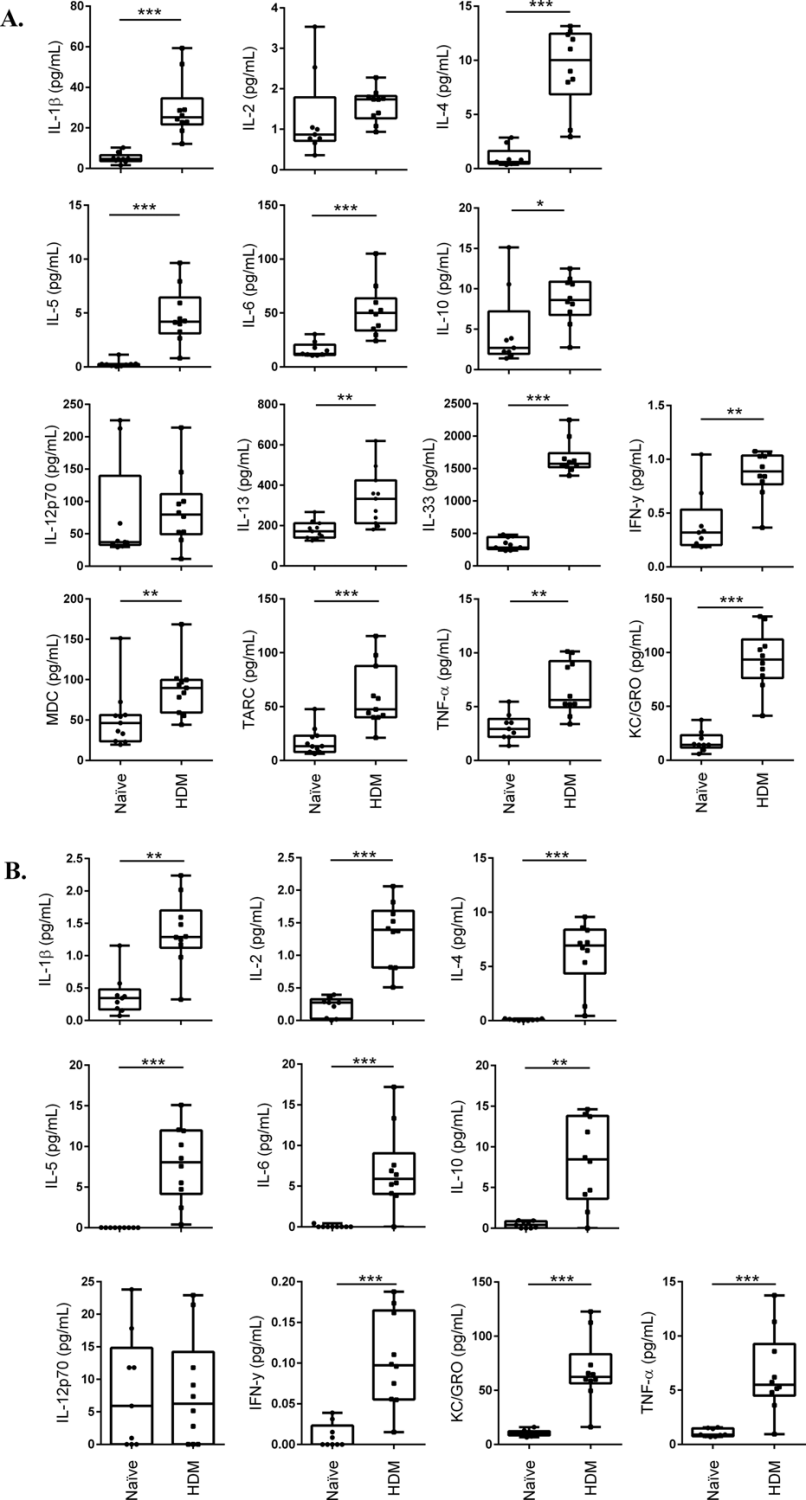
**HDM-induced cytokine profile in lung tissue lysates.** 8-10-week-old female Balb/c mice were challenged by intranasal administration of 35 µl of whole HDM extract (0.7 mg/ml) in saline, for two weeks (acute). (A) Lung tissue lysates (50 mg) and (B) BALF obtained from naïve (*n*=9) and HDM-challenged (*n*=10) mice were monitored for production of a panel of cytokines, 24 h after the last HDM challenge. Production of IFN-γ, IL-1β, IL-10, IL-12 p70, IL-2, IL-4, IL-5, IL-6, KC and TNF-α were monitored using the multiplex Meso Scale Discovery (MSD) platform, and, IL-33, MDC and TARC were monitored by ELISA. Results are shown as box plots with median line and statistical analyses was performed using the Mann–Whitney *U*-test (*P≤0.05, **P≤0.005, ***P≤0.0005).

We monitored the production of a panel of pro-inflammatory cytokines (IFN-γ, IL-1β, IL-10, IL-12 p70, IL-2, IL-4, IL-5, IL-6, KC and TNF-α) using a multiplex MSD platform in serum, BALF and lung tissue lysates. We further expanded this analyses to monitor the production of additional cytokines (IL-13, IL-33, MDC and TARC), transcripts of which were found to be upregulated after HDM-challenge in lung tissue lysates (Table 1), in the lung tissue lysates using ELISA. There was a significant increase in the production of cytokines IL-13, IL-10, TNF-α, IL-4, IL-33, IFN-γ, IL-1β, IL-5 and IL-6, and chemokines CCL17 (TARC), CCL22 (MDC) and KC, in lung tissues lysates of the HDM-challenged compared to allergen-naïve mice (Fig. 6A). Whereas, the abundance of cytokines IL-2 and IL-12p70 were similar in the lung tissue lysates obtained from HDM-challenged and allergen-naïve mice (Fig. 6A). To correlate the cytokine abundance observed in the lung tissue lysates in response to HDM challenge with that of those secreted in the airway milieu, we further measured cytokine and chemokine levels in BALF. Concentration of IFN-γ, IL-1β, IL-2, IL-4, IL-5, IL-6, IL-10 and TNF-α, and chemokine KC, were significantly higher in BALF of HDM-challenged compared to allergen-naïve mice (Fig. 6B). Notably, by contrast there was no change in abundance of cytokines and chemokines in serum of HDM-challenged mice compared to allergen-naïve mice (Fig. S4). These results suggested that the inflammatory response was localized to the lung tissue compartment in our HDM-challenge protocol.

## DISCUSSION

Murine models play a key role in dissecting the pathogenesis of human diseases, yet the potential assess such models using Systems Biology approaches have not been fully exploited. The HDM-challenge murine model of asthma results in a Th2-polarized bronchial inflammation, airway remodeling and epithelial damage similar to that seen in human asthma (Cates et al., 2004, 2007; Johnson et al., 2004). It is a pathophysiologically relevant model as HDM is the most prevalent allergen associated with asthma worldwide, up to 85% of asthmatics are HDM allergic (Gregory and Lloyd, 2011). A distinct advantage of the HDM-challenged murine model is that it does not induce tolerance, which is a problem associated with the murine models that employ an OVA-sensitization and repeated challenge protocol. However, previous studies have shown that outcomes measured using the HDM-challenged murine model can be variable, likely related to details of the HDM exposure protocol used (e.g. HDM concentration, timing and number of HDM exposure per week, and the time after the last HDM challenge when the outcomes are monitored). There are few studies that report objective biomarkers in HDM-challenged murine model that fully reveal the character of inflammation and its association with tissue repair and pathophysiological parameter of lung function (Ho et al., 2014; Koyama et al., 2015). Notably, a recent study by Koyama et al. performed (Koyama et al., 2015) transcriptomics (RNA sequencing) on lung tissues from a similar HDM-challenged murine model. However, they focused on the expression and function of only one biomarker from the transcripts upregulated in the HDM mice. Our study adds significantly to the previously published studies, as we have characterized a panel of distinct endpoints (biosignature) in lung tissue (the expression of 84 targeted genes) and BAL (a panel of inflammatory cytokines) from a HDM-challenged acute murine model. We have further performed a comprehensive network and pathways bioinformatics analyses to predict transcriptional regulators that may be contributing to the airway inflammation that characterizes this model. We speculate that the biosignature defined in this study will be useful endpoint surrogates for the human disease, especially to delineate molecular mechanisms underlying the progression from airway inflammation to tissue remodeling.

In this study, we comprehensively characterized various immune responses in the lung tissues, BALF and systemic blood, and monitored differential expression of a panel of 84 genes known to be associated with the disease process of allergy and asthma. We used an acute allergen challenge model featuring daily intranasal HDM challenge for five days per week for two weeks. Monitoring kinetics of cell infiltration in the lungs, we showed that neutrophil infiltration is most prominent 8 h after the last HDM challenge and subsequently declines (Fig. 1A). However, eosinophils and macrophage infiltration is seen beyond 8 h and steadily peaks 24 and 48 h after the last HDM-challenge in this model (Fig. 1A). The kinetics of neutrophil and eosinophil infiltration observed in this study was consistent with previous studies using the acute model of HDM-challenge (De Alba et al., 2010). We further demonstrated it was at least 24 h after the last HDM-challenge that there was a significant increase in all cell types in the HDM-challenged mice compared to allergen-naïve mice, albeit with decline in neutrophilic infiltration (Fig. 2B). These results suggested that 24 h after the last HDM-challenge was the most favorable time point to characterize molecular processes aligned with airway inflammation in this model. Therefore, in this study we characterized immune and transcriptional responses 24 h after the final HDM challenge. Our analyses of lung function, inflammation, and tissue changes demonstrate that the two week acute model of HDM-challenge results in AHR, increased circulating levels of allergen-specific immunoglobulins, and early signs of airway remodeling without subepithelial deposition of collagen. Thus, the model used in this study could be used to delineate the underlying mechanisms of airway inflammation that may be either independent or most likely preceding the process of airway remodeling.

Airway inflammation and AHR are the hallmarks of allergic asthma. The acute model described in this study indeed resulted in significant increase in maximum airway and tissue resistance, and tissue elastance to methacholine challenge, indicating AHR in HDM-challenged mice compared to allergen-naïve mice. These results were consistent with previous studies employing sensitization with HDM in animal models (Cates et al., 2004). It should be however noted that measurement of lung mechanics in this study showed significant robust increase in AHR to methacholine challenge when monitored 24 h (Fig. 2) as opposed to 8 h (Fig. S1), after the last HDM challenge. This data corroborated the selection of 24 h after the last HDM challenge to evaluate immune responses and transcriptional profile in this study. Furthermore, as eosinophil infiltration in the lung was seen after 8 h and beyond 24 h after the last HDM challenge (Fig. 1), our lung mechanics data also suggests that AHR may be related to eosinophil infiltration in the lungs. Furthermore, the bioinformatics analyses with IPA tool in this study revealed that 5 out of the 6 chemokines, and all three chemokine receptors upregulated in HDM lungs were associated with eosinophil migration, recruitment and eosinophilia (Fig. S5). This is consistent with a previous study (Crotty Alexander et al., 2013) demonstrating that negative regulation of eosinophil chemotaxis decreases lung inflammation and the development of AHR in asthma. However, the role of eosinophils remain poorly understood in the pathogenesis of human asthma, as clinical trials targeting eosinophil differentiation and survival produced equivocal results and failed to suppress AHR (Foster et al., 2008). Nevertheless, in the murine model discussed in this study, there appears to be a correlation between the kinetics of eosinophil infiltration in the lungs and the development of AHR following HDM challenge.

Similarly, there are conflicting data on the correlation of AHR with presence of circulating HDM-specific IgE antibodies in animal models. It has been previously demonstrated that AHR is not related to the presence of serum HDM-specific IgE (Tournoy et al., 2000). By contrast, other studies have demonstrated HDM-specific IgE and IgG1 plasma antibodies correlates with AHR (Cates et al., 2004; Lu et al., 2011), however these were in recall murine models of HDM-challenge. In the acute model discussed in this study, we have demonstrated the presence of HDM-specific IgE and IgG1, as well as significant increase in total IgG and IgE, in the serum of HDM-challenged mice compared to allergen-naïve mice (Fig. 4). Our result is in line with the immune response seen in the human disease where elevated levels of total IgE and presence of allergen-specific IgE has been correlated with allergic asthma (Schulman and Pohlig, 2015).

In this study, we demonstrated that despite significant cellular infiltration and increase in mucous producing goblet cells, there was no significant subepithelial collagen deposition in the airways of the HDM-challenged compared to allergen-naïve mice (Fig. 3). These results suggest that after two weeks of repeated HDM exposure the airways show early signs of tissue remodeling with increase in epithelial thickness and goblet cell hyperplasia, without fibrosis. This is consistent with previous studies suggesting that two-week exposure to HDM in murine models predominantly exhibit airway inflammation (Cates et al., 2004; Locke et al., 2007). However, the relationship between biological processes that contribute to airway inflammation and structural tissue changes has not been completely delineated (Bush, 2008). Previous studies have argued that the process of chronic inflammation in asthma may be partly distinct from those that result in tissue remodeling (Jeffery, 2001). Consequently, there is a need to explore therapeutics and alternate drug targets that can separately control the processes of airway inflammation and remodeling. The animal model described in this study may be thus beneficial to define the underlying mechanisms of airway inflammation that may be either independent of, or preceding the process of airway tissue remodeling in allergic asthma.

An impediment in defining the underlying mechanisms of allergic asthma has been limited in part due to the availability of specific biomarkers in animal models relevant to human asthma. Therefore in this study, we employed a quantitative PCR array to profile the expression of a panel of 84 genes known to contribute to the pathogenesis of allergy and asthma, primarily curated from human data. The most abundant and significantly upregulated mRNA in the DE genes from HDM lungs (Table 1) were molecules that have been previous shown to be induced in human asthma (Thai et al., 2005; Kulkarni et al., 2010; Louten et al., 2012; Bhakta et al., 2013) such as chloride channel calcium activated member-3 (*clca3*), eosinophil associated ribonuclease A family 11 (*ear11*) and mucin-5AC, those associated with allergic diseases (*il-13*, *il-13ra2*, *il-10* and *il-21*), and chemokines involved in cellular recruitment to the lungs (*ccl11*, *ccl12* and *ccl24*). The biological processes that were predicted to be significantly activated as a consequence of the DE genes were predominantly those associated with leukocyte recruitment especially eosinophils, AHR, secretion of mucus and cytokine signaling, all relevant to the pathophysiology of human allergic asthma. The DE genes identified to be upregulated following HDM challenge in this study confirms the relevance for the use of this model in delineating the pathophysiology and underlying mechanisms associated with human allergic asthma.

Bioinformatics analyses in this study defined upstream regulators predicted to be activated based on the DE genes (Table S2). Notably, among the predicted upstream regulators were IL-33 and the transcription factor retinoic acid-related orphan receptor α (RORA), both shown to significantly contribute to the pathogenesis of asthma in a recent genome-wide association study (Grotenboer et al., 2013). IL-33 is known to drive airway inflammation by engaging type 2 innate lymphoid cells (ILC2) and inducing the expression of cytokines IL-13 and IL-5. RORA is known to mediate the differentiation of ILC2, the primary cell targets of IL-33 (Scanlon and McKenzie, 2012). In this study we have shown that the mRNA transcripts (Table 1) and protein production of both IL-13 and IL-5 (Fig. 6) were significantly higher in the lungs of HDM-challenged mice compared to allergen-naïve mice. In addition, mRNA of IL1R1, which is a part of the IL-33 receptor complex expressed on innate lymphoid cells ILC2, was also upregulated after HDM challenge. These results suggest that the IL-33-RORA-ILC2 axis which prominently drives the production of IL-13 is activated in the model used in this study.

Among the upstream regulators predicted to be activated were also GATA-3 and IL-4 (Table S2). The transcription factor GATA-3 is known to promote the secretion of IL-4 from Th2 cells. We have shown that mRNA and protein production of IL-4 was induced in response to HDM challenged (Table 1; Fig. 6). Our results demonstrate that the IL-4-mediated canonical pathway driving Th2-inflammation and synthesis of IgE is also activated in this model. Recent studies have clearly defined the differential roles of IL-4 and IL-13 in allergic inflammation; IL-4 preferentially regulates Th2 cell function and IgE synthesis, whereas IL-13 mediates the pathophysiological processes that control mucus production, eosinophilia and AHR (Gour and Wills-Karp, 2015). Consistent with this, we have shown that HDM-challenged mice in this model have increased AHR, eosinophil infiltration, goblet cells, as well as increased Th2-cytokines and total and allergen-specific IgE. Taken together, our results indicate that both IL-4 and IL-13 mediated distinct downstream responses are induced in this model. However, IL-13 has also been shown to contribute to tissue remodeling (Gour and Wills-Karp, 2015) or fibrosis, which was not seen in this study. This is likely due to the duration of HDM exposure in this model, suggesting that further extending the duration of this model for a total of 5 to 8 weeks to incorporate prolonged exposure to HDM will likely result in airway remodeling and fibrosis. Nevertheless as described, this model demonstrates the hallmarks of airway inflammation associated with human asthma, employing the critical processes driven by both the cytokines IL-4 and IL-13.

In summary, we have demonstrated that the defined HDM-challenged model in this study results in the induction of a panel of biomolecules associated with human airway inflammation. We have systematically characterized immune responses, and provided a panel of objective regulatory and an endpoint biosignature that will be valuable to study disease progression in asthma using this murine model. The biosignature defined in this study will notably be valuable to define underlying molecular mechanisms of airway inflammation that precedes tissue remodeling in allergic asthma. Furthermore, as some of the defined biomolecules in this study do not yet have known functions in asthma, these will be useful to explore as novel candidates in the context of the pathophysiology of asthma. The comprehensive analyses of responses demonstrated the similarities between the murine model described in this study and the human disease, thus establishing the relevance of this model for preclinical studies in allergic asthma.

## MATERIALS AND METHODS

### Murine model of allergic asthma, acute HDM-challenge

The protocol used was approved by the University of Manitoba Animal Care Ethics Board. Seven week old female BALB/c mice were obtained from the Genetic Modeling of Disease Centre (University of Manitoba) and housed in the central animal care facility at the University of Manitoba. After a rest period of one week, mice were challenged for 5 consecutive days per week (day 1-5) for two weeks with intranasal administrations of 35 μl (0.7 mg/ml saline) of whole HDM protein extract (Greer Laboratories, Lenoir, NC, USA). Mice were sacrificed and outcomes were monitored 8, 24, 48, or 72 h after the last HDM challenge as indicated.

### Assessment of lung function

As described previously (Ma et al., 2013; Ryu et al., 2014; Sharma et al., 2014), mice were anesthetized with sodium pentobarbital and lung function was measured using a *flexi*Vent™ small animal ventilator (SCIREQ Inc, Montreal, QC, Canada) using Quick Prime-3 and Snapshot perturbations. After assessment of baseline measures of airway resistance (Rn), tissue resistance (G) and elastance (H) in response to nebulized saline, changes in these parameters in response to challenge with increasing concentrations of nebulized methacholine (3-50 mg/ml) were assayed.

### Sample collection and preparation

Mice were anesthetized using sodium pentobarbital followed by tracheostomy in which a cannula was inserted into the trachea and lung was washed twice, each time with 1 ml (total 2 ml) of cold saline to obtain bronchoalveolar lavage fluid (BALF) samples. BALF was centrifuged (1200 rpm, 10 min) to obtain cell-free supernatants. The supernatants were aliquoted and stored at −20°C for further use. Cell pellets were resuspended in 1 ml of saline and total cell count was performed using a hemocytometer. Cell differentials were performed using 100 μl of the resuspended pellet by cytospin using a modified Wright-Giemsa staining (Hema 3^®^ Stat Pack), counting eight image frames with a Carl Zeiss Axio Observer Z1 microscope, blinded by two different personnel. Blood was collected by severing the abdominal aorta, serum isolated, aliquoted and stored −20°C.

For RNA isolation, a specimen from the middle portion of the left lung was collected, stabilized in RNAlater^®^ (Life Technologies, Burlington, ON, Canada) at 4°C overnight and stored at −80°C until further use. Another set of lung tissue specimen from the right lung middle lobe was collected in Tissue Protein Extraction Reagent T-Per (Pierce; Thermo Fisher Scientific Inc, Rockford, IL, USA) containing protease inhibitor cocktail (Sigma Aldrich, Oakville, ON, Canada) and homogenized on ice using Cole-Parmer LabGEN 125 homogenizer (Cole-Parmer Canada Inc, Montreal, QC, Canada). The homogenates were centrifuged (10,000×***g***) to clarify lysates. Protein amount in the tissue lysate was quantified with bicinchoninic acid (BCA) Protein assay (Pierce). The lysates were aliquoted and stored at −80°C.

### Gene expression profiling using quantitative real-time PCR array

Lung tissues collected from HDM-challenged and naïve mice, 24 h after the final HDM challenge, were homogenized in RNA lysis buffer using the Cole-Parmer LabGEN 125 homogenizer. Total RNA was isolated using the RNeasy kit (Qiagen Inc, ON, Canada). A RT^2^ Profiler™ PCR Array (SABiosciences, Qiagen) was employed using ABI 7300 Quantitative Real time PCR system to profile the expression of 84 targeted genes related to in allergy and asthma (RT^2^ Profiler™ PCR Array Mouse Allergy & Asthma, Catalog No. PAMM-067ZA), as specified by the manufacturer. Data quality control and analyses to calculate fold changes was performed using the PCR Array data analysis software as per the manufacturer's instructions (Qiagen, data analysis center). Briefly, data analysis was based on the ΔΔC_T_ method with normalization using three housekeeping genes (β-actin, β-glucuronidase and Hsp90ab1). Network and pathway analysis was performed using the Ingenuity^®^ Pathway Analysis (IPA) tool (Qiagen Inc).

### Multiplex cytokine analyses

Serum, BALF and lung tissue lysates were monitored for the production of a panel of murine cytokines and chemokines with the V-plex Pro-Inflammatory Panel 1 (IFN-γ, IL-1β, IL-10, IL-12p70, IL-2, IL-4, IL-5, IL-6, KC and TNF-α), catalog #K15048D-1, using the multiplex Meso Scale Discovery (MSD) platform (Meso Scale Discovery, Rockville, MD, USA) as per the manufacturer's instructions. Data was analyzed using the Discovery Workbench 4.0 software (Meso Scale Discovery). In addition, specific antibody pairs (R&D Systems Inc, Minneapolis, MN, USA) were used to monitor the production of IL-33 (catalog #DY3626), MDC (catalog #MCC220) and TARC (catalog #MCC170) by ELISA, as per the manufacturer's instructions. Serum and BALF samples were diluted 1:2 for MSD analysis, and used undiluted for ELISA. 50 mg of total protein was used from each lung tissue lysate for cytokine evaluation. Serial dilutions of the recombinant murine cytokines were used to establish a standard curve for the evaluation of the cytokine concentrations by ELISA.

### Detection of total and HDM-specific antibodies

Serum concentrations of total IgE (catalog #88-50460) and IgG (catalog #88-50400) were assessed by Ready-Set-Go^®^ kits (eBioscience Inc., San Diego, CA, USA) as per the manufacturer's instructions. Serum samples were diluted 1:50 for total IgE and 1:50,000 for total IgG, in PBS containing 1% BSA (w/v). An indirect ELISA method was used to assess the HDM-specific IgE and IgG1 levels in serum samples. Briefly, 100 µl per well of 10 µg/ml of HDM extract (Greer Laboratories) in PBS was used to coat Costar™ 96-well flat-well high-binding plates (Thermo Fisher Scientific) overnight at 4°C. The plates were blocked with 3% BSA (w/v) in PBS overnight at 4°C. Serum samples used for detection of HDM-specific IgE were precleared by incubating 1:1 with Protein G Sepharose beads overnight at 4°C. Serum samples (50 µl per well) were incubated overnight at 4°C. Biotin conjugated goat anti-mouse IgE or goat anti-mouse IgG1 (Southern Biotech, Birmingham, AL, USA) were used as secondary detection antibodies (1:5000 dilution in PBS containing 1% BSA). Avidin-HRP (eBioscience) and TMB (Thermo Scientific) were used to yield a colorimetric reaction. Reaction was stopped with 2 N H_2_SO_4_, and absorbance recorded using a BioTek Synergy 4 Microplate reader.

### Histology

As previously described (Zhu et al., 2011; Ryu et al., 2014), lungs were inflated *in situ* through a tracheal cannula with ∼1 ml (20 cm H_2_O) of 10% v/v formalin and the trachea was sealed. The whole lung was removed, transferred into formalin for 24 h (room temperature) and subsequently transferred into PBS. Whole lung was dehydrated using ethanol and xylene, embedded in paraffin and 6 µm sections were obtained. Paraffin embedded lung sections were stained with hematoxylin and eosin (H&E) to enumerate inflammatory cell infiltration and epithelial thickening. Using the Carl Zeiss Zen software, basement membrane of airways was outlined and area inside the contoured space (A µm^2^) and perimeter of the outline was measured. A second contour was drawn around the outer edge of the epithelium and area inside was recorded as airway space (B µm^2^). The ratio of the difference between the two areas (A−B µm^2^) and length of basement membrane (perimeter µm) was determined as mean epithelial thickness (µm) per 1 µm of basement membrane. Periodic acid Schiff (PAS) stain was used to assess airway epithelial goblet cell abundance and or Picrosirius Red was used to assess collagen deposition.
